# Pyrosequencing and Taxonomic Composition of the Fungal Community from Soil of *Tricholoma matsutake* in Gyeongju

**DOI:** 10.4014/jmb.2103.03021

**Published:** 2021-03-26

**Authors:** Minji Jeong, Doo-Ho Choi, Woo-Jae Cheon, Jong-Guk Kim

**Affiliations:** 1 Department of Life Sciences and Biotechnology, Kyungpook National University, Daegu 41566, Republic of Korea; 2 Department of Forest Environment, Gyeongsangbuk-do Forest Environment Research Institute, Gyeong-ju 38174, Republic of Korea

**Keywords:** Fungal community, *Tricholoma matsutake*, metagenomics

## Abstract

*Tricholoma matsutake* is an ectomycorrhizal fungus that has a symbiotic relationship with the root of *Pinus densiflora*. Soil microbial communities greatly affect the growth of *T. matsutake*, however, few studies have examined the characteristics of these communities. In the present study, we analyzed soil fungal communities from Gyeongju and Yeongdeok using metagenomic pyrosequencing to investigate differences in fungal species diversity, richness, and taxonomic composition between the soil under *T. matsutake* fruiting bodies (Sample 2) and soil where the fairy ring of *T. matsutake* was no longer present (Sample 1). The same spot was investigated three times at intervals of four months to observe changes in the community. In the samples from Yeongdeok, the number of valid reads was lower than that at Gyeongju. The operational taxonomic units of most Sample 2 groups were less than those of Sample 1 groups, indicating that fungal diversity was low in the *T. matsutake*dominant soil. The soil under the *T. matsutake* fruiting bodies was dominated by more than 51% *T. matsutake*. From fall to the following spring, the ratio of *T. matsutake* decreased. Basidiomycota was the dominant phylum in most samples. G-F1-2, G-F2-2, and Y-F1-2 had the genera *Tricholoma*, *Umbelopsis*, *Oidiodendron*, *Sagenomella*, *Cladophialophora*, and *Phialocephala* in common. G-F1-1, G-F2-1, and Y-F1-1 had 10 genera including *Umbelopsis* and *Sagenomella* in common. From fall to the following spring, the amount of phyla Basidiomycota and Mucoromycota gradually decreased but that of phylum Ascomycota increased. We suggest that the genus *Umbelopsis* is positively related to *T. matsutake*.

## Introduction

*Tricholoma matsutake*, known as the pine mushroom, is an ectomycorrhizal fungus that has a symbiotic relationship with the root of *Pinus densiflora* trees. *T. matsutake* is used medicinally and is also one of the most famous food items in Northeast Asia because of its unique flavor and taste [1-6]. In South Korea, the yield of *T. matsutake* is changeable because environmental factors such as temperature and precipitation greatly affect its growth [7]. For this reason, studies have examined the possibility of creating a stable supply of the pine mushroom, but currently, artificial cultivation is not feasible. The effect of environmental factors (climate, soil condition, microbial community, and others) on its growth and interactions between *T. matsutake* and *P. densiflora* requires further research [8-10]. Soil microbial communities, in particular, are important to the mycelial growth of *T. matsutake*, however, few studies on the characteristics of the communities have been conducted [11-14].

Mycorrhizal fungi are an important group of fungi in the soil ecosystem. They have a mutualistic relationship with living plants, exchanging nutrients and some important compounds [15-19]. The major group of mycorrhizal fungi is the ectomycorrhizae (ECM) fungi group. ECM fungi, including *T. matsutake*, considerably affect the growth of living plants by colonizing close by the rootlets of living plants. ECM fungi form ECM with roots of host plants and have advantageous symbiotic relationships with them. ECM are symbiotic organs between soil and roots and help the trees absorb water and nutrients, receiving carbohydrates from them in exchange to grow mycelium [20-22]. The fungus *Arthrinium phaeospermum* promotes mycorrhiza formation, similar to the effect of mycorrhization helper bacteria [23]. *T. matsutake* is an ectomycorrhizal symbiont that is important to the growth of host plant seedlings [24]. In addition, some bacteria and fungi have positive effects on *T. matsutake* [25]. The soil fungal community is positively influenced by plant species richness and diversity [26]. The interaction between plants and mycorrhizal fungi plays a key role in the circulation of nutrients and the balance of the ecosystem [27-30]. Therefore, the study of soil fungal communities near *T. matsutake* should be conducted constantly and persistently.

Next-generation sequencing (NGS) enables the rapid analysis of massive amounts of DNA sequences. It also permits the analysis of DNA sequences from microorganisms that are hard to cultivate [31-34]. Pyrosequencing is one of the sequencing approaches for NGS and has facilitated the study of mass and diverse microorganisms from soil samples. For this study, we used the Illumina MiSeq sequencing platform, which uses the sequences of ribosomal DNA as DNA barcodes to classify the soil fungi rapidly.

Here, we statistically analyzed the characteristics of soil fungal communities from two regions using metagenomic pyrosequencing with barcode to investigate diversity, species richness, and relative abundance or taxonomic composition of the communities in areas where *T. matsutake* fruiting bodies are present [35].

## Materials and Methods

### Collection of Soil Samples

We collected two soil samples each in pine forests in October 2017 in Gyeongju and in November 2017 in Yeongdeok, South Korea. Two samples were also collected in October 2018 and in February and June 2019 at Gyeongju. Immediately after harvesting the fruiting body of *T. matsutake*, we took soil samples of 10~15 cm depth and 2 cm in diameter from right beneath the fruiting body using a sterilized soil sampler. These samples were called G-F1-2 (2017, Gyeongju), G-F2-2 (2018, Gyeongju), and Y-F1-2 (2017, Yeongdeok) (Table 1). About 25~30cm from the fairy ring, we once again collected soil samples of 10~15 cm depth and 2 cm in diameter. These samples were called G-F1-1 (2017, Gyeongju), G-F2-1 (2018, Gyeongju), and Y-F1-1 (2017, Yeongdeok). The same spots sampled in the fall of 2018 were re-sampled four months later, in February 2019, and these samples were named G-W-2 and G-W-1. They were re-sampled again four months later, in June 2019, and these samples were named G-S-2 and G-S-1. So, ‘-1’ was attached to the end of the name for the samples expected to be *T. matsutake*-minor (Sample-1) and ‘-2’ was attached to the samples expected to be *T. matsutake*-dominant (Sample-2).

GPS coordinates of the sampling sites were as follows:
• G-F1: 35°47'22.1" N, 129°14'06.9" E
• Y-F1: 36°28'39.1" N, 129°22'12.0" E
• G-F2, G-W, G-S: 35°47'20.0" N, 129°14'09.0" E

### DNA Extraction and Pyrosequencing

Each 10 g of soil samples was used for isolation of metagenomic DNA using the FastDNA SPIN Kit for Soil (MP Biomedicals, USA) to conduct the pyrosequencing. Polymerase Chain Reaction (PCR) targeting the ITS2 region was used to amplify isolated chromosomal DNA. The sequences of the forward primer were 5′-AATGATACGGCGACCACCGAGATCTACAC-XXXXXXXX-TCGTCGGCAGCGTC-AGATGTGTATAAGAGACAG-GCATCGATGAAGAACGCAGC-3′. The sequences of the reverse primer were 5′- CAAGCAGAAGACGGC ATACGAGAT-XXXXXXXX-GTCTCGTGGGCTCGG-AGATGTGTATAAGAGACAGTCC-TCCGCTTATTGA TATGC-3′. “X” indicates that the barcode sequence differed between samples. A PTC-200 Peltier Thermal Cycler (MJ Research, USA) was used to conduct the PCR. For separation of PCR products, identical amounts of PCR products from each sample were used by agarose gel electrophoresis. The products of PCR were purified using the CleanPCR (CleanNA, Netherlands) and the quantification of the purified PCR products was performed with a Quant-iT PicoGreen dsDNA Assay Kit (Invitrogen, USA). PCR products longer than 300 bp were purified, and an Agilent 2100 Bioanalyzer (Agilent Technologies, USA) was used to analyze the base sequence of the DNA fragments [36]. In addition, the Illumina MiSeq sequencing platform (Illumina, USA) was used for the pyrosequencing analysis conducted by Chunlab Inc. (Korea), following the instructions supplied by the manufacturer [37]. Pyrosequencing reads data were submitted to the EMBL-EMI database (www.ebi.ac.uk) under accession number PRJEB42318 (primary) and ERP126157 (secondary).

### Statistical Data Analysis and Taxonomic Identification

The pyrosequencing raw reads were processed by using the barcode sequences. The fusion primers with barcode and low-quality reads were trimmed and removed using Trimmomatic 0.32 [38]. We used CLcommunity software (Chunlab Inc.) for statistical analysis. To identify operational taxonomic units (OTUs) at 97% sequence similarity [39], the CD-HIT program was used. The Mothur platform [40, 41] was used to calculate the rarefaction curves and diversity indices. For taxonomic composition and relative abundance, random sample subsets were generated with the lowest number of reads. Conditionally, *T. matsutake* was excluded for analysis of relative abundance and taxonomic composition.

## Results and Discussion

### Pyrosequencing Results and Statistical Data Analysis

**Fig. 1:**
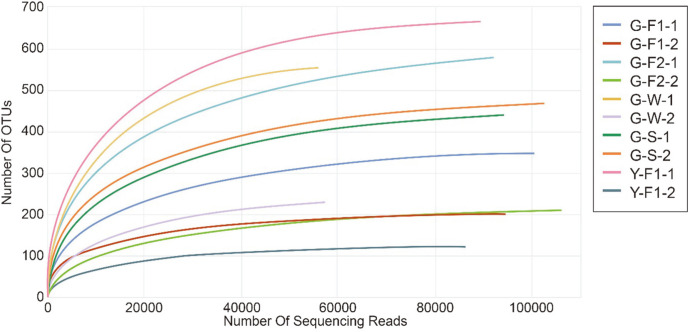
Rarefaction curves for operational taxonomic units (OTUs) of fungi from all soil samples at Gyeongju and Yeongdeok. OTUs were clustered at 97% similarity with CD-HIT. This shows the rate of increase in the number of OTUs (species) with the number of acquired sequences. The curves of *T. matsutake*-dominant soil samples were in low positions. This implies that the fungal diversity is low in *T. matsutake*-dominant soil samples (X-axis: the number of sequencing reads; Y-axis: the number of OTUs).

For fungal communities, the total number of valid reads after preprocessing was 881,125 from all eight samples (Table 2). The number of valid reads of G-F2-2 was greatest, at 106,048, followed by G-S-2 at 102,565, G-F1-1 at 101,347, G-F1-2 at 94,695, G-S-1 at 94,346, G-F2-1 at 92,141, Y-F1-1 at 89,952, Y-F1-2 at 86,224, G-W-2 at 57,412, and G-W-1 at 56,395. Overall, there were a large enough number of reads to analyze the microbial communities. Obviously, with fewer reads in the winter samples, the overall number of surviving fungi was reduced. However, the spring samples showed that the number of reads increased again and the ecosystem of soil microbes recovered. In the sample from Yeongdeok, the number of valid reads was slightly lower than that from Gyeongju. The OTUs of Y-F1-1 were the highest, at 664, and those of Y-F1-2 were the lowest, at 124. These two samples showed the biggest difference, at 540. On the other hand, the G-S-1 and G-S-2 samples were 439 and 469, respectively, showing 30 different results. The OTUs of most Sample 2 groups were lower than those of Sample 1 groups. Naturally, the Shannon index, which is one of the most important diversity indices, was similar to the number of OTUs. For the most part, the number for Sample 2’s was lower. In particular, G-F2-1 and G-F2-2 showed a significantly large difference. The Shannon index measures species richness and evenness, which means that the higher the number, the greater the diversity of the community. Therefore, fungal diversity was low in the soil samples in which *T. matsutake* was dominant. These results are also shown in the Simpson index results, which are presented together to produce more accurate results. Furthermore, the rarefaction curves supported the results above (Fig. 1). In the rarefaction curves, the number of OTUs increased with the number of reads, and the species richness was shown. This is consistent with previous studies [12, 13, 25] showing that fungal diversity is low in *T. matsutake*-dominant soil samples. This confirms that the mycelia of *T. matsutake* repress the growth of other fungi and are dominant in the soil fungal community.

### Comparison of Fungal Communities: Composition of *T. matsutake*

First, for analysis of taxonomic composition and relative abundance, random sample subsets were generated with the lowest number of reads: 56395 (G-W-1).

The composition of *T. matsutake* was analyzed first, followed by the composition of the entire fungal community, and finally, a seasonal comparison was conducted (Table 3). As expected, the spot where *T. matsutake* was present was clearly dominated by *T. matsutake* at more than 51%. The difference between where *T. matsutake* was present or not present was greater than 50%, except in the G-S samples. This indicates that when the fruiting body of *T. matsutake* is formed, *T. matsutake* is definitely a dominant species in that spot. From G-F2-2 to G-W-2 and to G-S-2, the ratio decreased. From G-F2-2 in fall to G-W-2 in winter, the difference was 3.94%, which was a slight drop. But from G-W-2 to G-S-2, the difference was 23.08%. The difference in G-S where *T. matsutake* was present or not was 13.19%, which was less than the difference in other samples. In addition, the somewhat low share in the G-S-2 sample, at 52.11%, seems to have naturally decreased as the growth of other fungi became more active with the rise in temperature.

### Comparison of Fungal Communities by Region

**Fig. 2:**
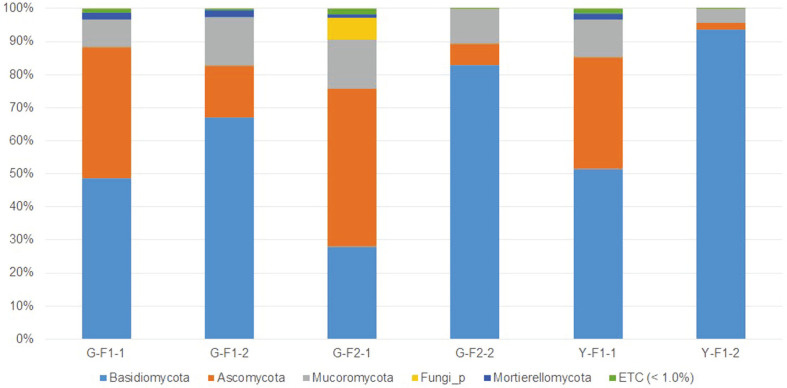
Taxonomic composition at the phylum level from G-F1-1, G-F1-2, G-F2-1, G-F2-2, Y-F1-1, and Y-F1- 2 samples. Samples of Gyeongju and Yeongdeok collected in autumn were compared. Fungal phyla with a relative abundance greater than 1% in at least one of the samples are shown and phyla less than 1% were shown as ETC. Basidiomycota was more abundant in Sample-2 (under fruiting bodies of *T. matsutake*) than Sample-1 (far from the fairy ring) regardless of regions and was significantly dominant in Y-F1-2 (G-F1: October 2017, G-F2: October 2018, and Y-F1: October 2017).

First, we conducted an analysis at the phylum level of the taxonomic composition of soil samples from G-F1-1, G-F1-2, G-F2-1, G-F2-2, Y-F1-1, and Y-F1-2. A total of five phyla were identified (>1%): Basidiomycota, Ascomycota, Mucoromycota, Fungi_p (phylum name unknown), and Mortierellomycota (Fig. 2). Including *T. matsutake*, Basidiomycota was the dominant phylum in G-F1-1 (48.49%), G-F1-2 (66.97%), G-F2-2 (82.84%), Y-F1-1 (51.42%), and Y-F1-2 (93.59%). Ascomycota was dominant only in G-F2-1 (47.64%). The ratio of Mucoromycota was G-F1-1 (8.22%), G-F1-2 (14.82%), G-F2-1 (15.02%), G-F2-2 (10.65%), Y-F1-1 (11.63%), and Y-F1-2 (4.18%). The ratio of Fungi_p was 6.42% in G-F2-1 and 0.03% in G-F2-2. The percentage of Mortierellomycota was as follows: G-F1-1 (2.07%), G-F1-2 (2.04%), G-F2-1 (1.11%), G-F2-2 (0.03%), Y-F1-1 (1.85%), and Y-F1-2 (0.05%). Excluding *T. matsutake*, Basidiomycota was the dominant phylum in G-F1-1 (48.49%), Y-F1-1 (51.42%), and Y-F1-2 (86.57%), and Ascomycota was dominant in G-F1-2 (36.87%) and G-F2-1 (47.70%). To determine the taxonomic composition of fungi except *T. matsutake* and to analyze the effects of *T. matsutake* on the composition of other fungi, an additional comparative analysis was performed after excluding the reads of the above analyzed *T. matsutake*. Excluding *T. matsutake*, the ratio of Basidiomycota was lower in the regions where *T. matsutake* grew than in the regions where it did not grow (G-F1-1 > G-F1-2, G-F2-1 > G-F2-2). In the Yeongdeok samples, the ratio was Y-F1-1 < Y-F1-2 because of the unclassified genera *Ramaria* of Basidiomycota in Y-F1-2, which is mentioned again at the genus level.

At the class level, a total of nine fungal classes found to represent more than 1% of all the classes were identified: Agaricomycetes, Umbelopsidomycetes, Eurotiomycetes, Leotiomycetes, Dothideomycetes, Sordariomycetes, Mortierellomycetes, Fungi_c (class name was unknown), and GS25 (unnamed). We focused on the classes of the relatively abundant phyla Basidiomycota, Ascomycota, Mucoromycota, and Mortierellomycota. In phylum Basidiomycota, we found Agaricomycetes in G-F1-1 (47.99%), G-F1-2 (65.95%), G-F2-1 (26.23%), G-F2-2 (80.12%), Y-F1-1 (46.39%), and Y-F1-2 (93.17%). Phylum Mucoromycota was represented by Umbelopsidomycetes: G-F1-1 (8.22%), G-F1-2 (14.82%), G-F2-1 (14.33%), G-F2-2 (10.60%), Y-F1-1 (11.63%), and Y-F1-2 (4.18%). Class Umbelopsidomycetes was the second most dominant class in all Sample 2’s after class Agaricomycetes, including *T. matsutake*. For phylum Ascomycota, we found Eurotiomycetes: G-F1-1 (25.05%), G-F1-2 (2.31%), G-F2-1 (11.49%), G-F2-2 (2.32%), Y-F1-1 (8.51%), Y-F1-2 (0.56%); Leotiomycetes: G-F1-1 (3.09%), G-F1-2 (5.15%), G-F2-1 (20.92%), G-F2-2 (2.01%), Y-F1-1 (14.11%), and Y-F1-2 (1.22%); and Dothideomycetes: G-F1-1 (10.70%), G-F1-2 (5.72%), G-F2-1 (6.88%), G-F2-2 (1.84%), Y-F1-1 (5.82%), and Y-F1-2 (0.13%). The classes in phylum Ascomycota had a tendency to show a smaller percentage in Sample 2’s than in Sample 1’s. For phylum Mortierellomycota, we found Mortierellomycetes: G-F1-1 (2.07%), G-F1-2 (2.04%), G-F2-1 (1.11%), G-F2-2 (0.03%), Y-F1-1 (1.85%), and Y-F1-2 (0.05%). Mortierellomycetes was present at a lower percentage in Sample 2’s than in Sample 1’s.

At the genus level, the top 10 most abundant genera were *Tricholoma*, *Umbelopsis*, *Ramaria*, *Tylospora*, *Sagenomella*, *Cenococcum*, *Hydnum*, *Oidiodendron*, *Russula*, and *Sebacina* (Table 4) in G-F1-1, G-F1-2, G-F2-1, G-F2-2, Y-F1-1, and Y-F1-2. In *T. matsutake*-dominant G-F1-2 and G-F2-2 samples, *Tricholoma*, *Umbelopsis*, *Cenococcum*, *Oidiodendron*, *Sagenomella*, *Cladophialophora*, *Penicillium*, *Rhizopogon*, and *Phialocephala* were the only genera that existed in common (> 0.1%). In both *T. matsutake*-minor G-F1-1 and G-F2-1, the genera *Sagenomella*, *Umbelopsis*, *Cenococcum*, *Hydnum*, *Russula*, *Oidiodendron*, *Tricholoma*, *Penicillium*, *Sistotrema*, *Phialocephala*, *Talaromyces*, Fungi_uc (unclassified), Herpotrichiellaceae_uc, and Botryosphaeriaceae_uc (>0.1%) were present.

Compared to the fungal community from Yeongdeok, Y-F1-1 shared *Tylospora* with G-F1-1 at 10.27% and 22.24%, respectively, while none were present in G-F2-1. The genus *Tylospora fibrillosa* is a nitrogen (N)-tolerant ectomycorrhizal fungus and contributes to the generation of N_2_O [42]. Furthermore, the genus *Hydnum* in the sample from G-F1-1 and G-F2-1 was not present in Y-F1-1. All species of *Hydnum* are ectomycorrhizae that coexist with trees such as the Pinaceae and Fagales in Europe [43]. *Sebacina* (10.90%), *Pseudotomentella* (9.60%), *Cadophora* (7.61%), *Suillus* (4.64%) were found in Y-F1-1, but in G-F1-1 and G-F2-1, they were either present at very low levels or not present. On the other hand, in Y-F1-1, *Umbelopsis* (11.43%), *Sagenomella* (1.06%), *Cenococcum* (2.09%), *Russula* (6.19%), and *Oidiodendron* (3.32%) existed in common with G-F1-1 and G-F2-1. Among *T. matsutake*-dominant samples, G-F1-2, G-F2-2, and Y-F1-2 showed that *Tricholoma*, *Umbelopsis*, *Oidiodendron*, *Sagenomella*, *Cladophialophora* and *Phialocephala* were the only genera that existed in common (>0.1%) (Table 4). Most of genus *Tricholoma* (G-F1-2, 58.01%; G-F2-2, 79.18%; Y-F1-2, 52.42%) was *T. matsutake*. The genus *Umbelopsis* was similar but differed by ratio at 14.50%, 10.60%, and 1.68%, which was significantly lower only in Y-F1-2. Similarly, the genus *Oidiodendron* was 4.24% in G-F1-2, 1.17% in G-F2-2, but 0.48% in Y-F1-2. The genus *Sagenomella* was 0.30% and 1.75%, but 0.38% in Y-F1-2. On the other hand, the genus *Ramaria* comprised 38.59% only in Y-F1-2 but was not present in G-F1-2 or G-F2-2. In *T. matsutake*-minor G-F1-1, G-F2-1, and Y-F1-1, the genera *Umbelopsis*, *Sagenomella*, *Russula*, *Cenococcum*, *Oidiodendron*, *Penicillium*, *Talaromyces*, *Phialocephala*, Herpotrichiellaceae_uc and Fungi_uc (>0.1%) were present (Table 4). In particular, the genus *Russula* was significantly lower in Sample 2, but was 4.84%, 7.34%, and 6.19% in Sample 1.

In our results, *Umbelopsis* was the most dominant genus, following genus *Tricholoma*, in most *T. matsutake*-dominant samples. *Umbelopsis* has been reported on frequently in other studies on the fungal community of *T. matsutake*. Ogawa and Kawai announced that metabolites produced by *Umbelopsis* promoted *T. matsutake*'s growth [44]. Also, *Umbelopsis* was isolated from the *P. densiflora* rootlet colonized by *T. matsutake* [45]. A previous study showed that genus *Umbelopsis* was remarkably more abundant in *T. matsutake*-dominant soil than in *T. matsutake*-minor soil; in comparison, *Mortierella* was outstandingly more abundant in *T. matsutake*-minor soil [25]. Oh and his colleagues reported that some bacteria isolated from the fruiting body of *T. matsutake* increased the growth of *Umbelopsis*, suggesting a strong link between *T. matsutake* and *Umbelopsis* [46]. *Umbelopsis* quickly grows into a nutrient-rich environment of fresh litters in the fall, when *T. matsutake* fruiting body is produced. *Umbelopsis* is also known as one of the common fungi associated with seedlings and healthy roots of pine tree [47, 48]. Tominaga discovered the relationship between *T. matsutake* and *Umbelopsis* by separating *Umbelopsis* from both mycorrhizae and the fruiting body of *T. matsutake* [49].

However, in the *T. matsutake*-minor G-F2-1 and Y-F1-1 samples, *Umbelopsis* was the most dominant genus at 14.29% and 11.47% each, respectively. Ogawa discovered that the zone of shiro at which fruiting body of *T. matsutake* forms *Mortierella* sp. (probably *Umbelopsis*) increased, and at the end of the shiro, *Mortierella* sp. and the other root fungi were commonly abundant [50]. Thus, we suggested that the sampling spots of G-F2-1 and Y-F1-1 could be near the end of the shiro and *Umbelopsis* was abundant in that spot. The interaction between *Umbelopsis* and *T. matsutake* remains uncertain and thus further study is needed to determine which mechanisms are interacting with them.

On the other hand, in our samples, there were several genera that were lower in Sample 2 than in Sample 1. The ratios of these genera were as follows: *Sagenomella* (G-F1-1, 20.95% and G-F1-2, 0.30%; G-F2-1, 2.97% and G-F2-2, 1.75%; Y-F1-1, 1.06% and Y-F1-2, 0.38%), *Cenococcum* (10.49% and 4.86%; 5.55% and 1.83%; 2.09% and 0.04%), *Russula* (4.84% and 0.00%; 7.34% and 0.31%; 6.19% and 0.00%), and *Penicillium* (1.65% and 0.70%; 2.84%and 0.11%; 2.96% and 0.03%).

For comparison of species comprising less than 1% of the community, only this number was compared with samples before normalization. Vaario and colleagues found that *Tomentellopsis* sp., *Cortinarius biformis*, *Tylospora* sp., *tomentellopsis submollis*, and *Trichoderma viride* were positively correlated with the presence of *T. matsutake* in soil above the fairy ring of *T. matsutake* [14]. Additionally, species *Piloderma* sp. 4, *Clavulina* cf. *amethystine*, and *Piloderma* sp. 3 were positively correlated with the presence of *T. matsutake* in soil in the fairy ring of *T. matsutake*. However, in our analysis, each of these species were present at a significantly low level (< 0.1%), and they existed in both *T. matsutake*-dominant and -minor soil. Thus, it is hard to say that they had a positive relationship. Furthermore, *Tylospora*_uc (unclassified into sublevel) comprised 22.24% of G-F1-1 and 10.27% of Y-F-1 but 0.00% in all *T. matsutake*-dominant samples, which suggests that they were not in a positive relationship. In comparison with the number of reads, there was only one species, *Aspergillus cervinus*, which was present in G-F1-2 (6 reads) and G-F2-2 (7 reads) but not in G-F1-1 and G-F2-1. *Aspergillus cervinus* was originally isolated from African soil [51] and generates the quinol terremutin and 3,6-dihydroxy-2,5-toluquinone [52, 53]. There were no species present in G-F1-2, G-F2-2, and Y-F1-2 that were not present in G-F1-1, G-F2-1, and Y-F2-1. However, in G-F1-2, G-F2-2, and Y-F1-2, there were 14 species in common with their number of reads in Table 5.

### Comparison of Fungal Communities by Season

**Fig. 3:**
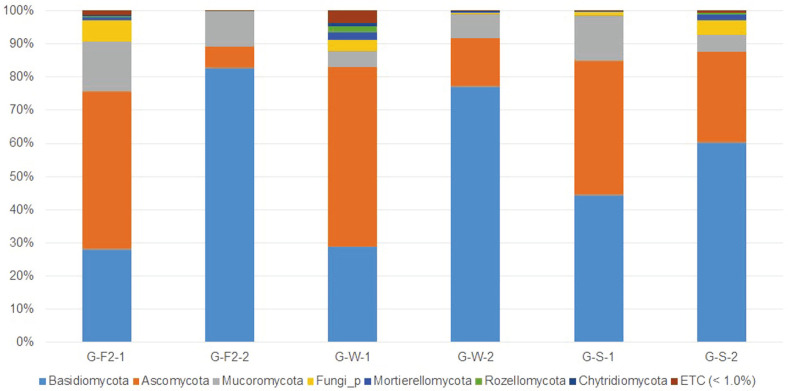
Taxonomic composition at the phylum level from the G-F2-1, G-F2-2, G-W-1, G-W-2, G-S-1, G-S-2 samples. Samples of only Gyeongju collected in autumn, winter, spring were compared. Fungal phyla with a relative abundance greater than 1% in at least one of the samples are shown and phyla less than 1% were shown as ETC. From G-F2-2 to G-W-2 to G-S-2, the ratio of the phylum Basidiomycota gradually dropped (G-F2: October 2018, G-W: February 2019, and GS: June 2019, -1: far from the fairy ring, -2: under fruiting bodies of *T. matsutake*).

A total of seven distinct fungal phyla were found to represent more than 1% of the total phyla in the G-F2, G-W, and G-S samples (Fig. 3). From G-F2-2 to G-W-2 to G-S-2, the ratio of the phylum Basidiomycota gradually dropped from 82.84% to 77.01% to 60.28%, respectively. Additionally, the phylum Mucoromycota was reduced from 10.65% to 7.29% to 5.17%. In contrast, the level of phylum Ascomycota was increasingly higher at 6.37%, 14.70%, and 27.29%, respectively. Likewise, other phyla showed a small difference in ratio, including Fungi_p (0.03%, 0.37%, and 4.30%), Mortierellomycota (0.03%, 0.14%, and 1.72%), Rozellomycota (0.02%, 0.21%, and 0.52%), and Chytridiomycota (0.01%, 0.09%, and 0.17%).

A total of 11 fungal classes representing more than 1% of all the classes in each sample were identified: Agaricomycetes, Leotiomycetes, Eurotiomycetes, Umbelopsidomycetes, Dothideomycetes, Sordariomycetes, Fungi_c, GS25, Mortierellomycetes, Lecanoromycetes, and Rozellomycotina cls *Incertae sedis* (unknown). We focused on the classes of the relatively abundant phyla Basidiomycota, Ascomycota, Mucoromycota, and Mortierellomycota. For phylum Basidiomycota, Agaricomycetes were present as follows: G-F2-2, 80.12%; G-W-2, 76.18%; and G-S-2, 59.15%. From G-F2-2 to G-W-2 to G-S-2, the ratio of Agaricomycetes decreased. For phylum Ascomycota, the ratios were as follows, respectively: Leotiomycetes: 2.01%, 3.57%, and 10.81%, Eurotiomycetes: 2.32%, 9.14%, and 11.31%; Dothideomycetes: 1.84%, 1.23%, and 1.12%; Sordariomycetes: 0.08%, 0.58%, and 3.45%; Lecanoromycetes: 0.01%, 0.02%, and 0.03%. From G-F2-2 to G-W-2 to G-S-2, Leotiomycetes, Eurotiomycetes, Sordariomycetes, and Lecanoromycetes increased; in contrast, Dothideomycetes decreased. For phylum Mucoromycota, we found Umbelopsidomycetes: 10.60%, 7.23%, and 4.88%. From G-F2-2 to G-W-2 to G-S-2, the proportion of this class decreased. For phylum Mortierellomycota, we found Mortierellomycetes at 0.03%, 0.14%, and 1.72%, and the percentages were increasing.

At the genus level, *Oidiodendron* of Leotiomycetes increased from 1.17% to 2.21% to 3.25% in G-F2-2, G-W-2, and G-S-2, respectively. Likewise, the genus Hyaloscyphaceae_g (genus name unknown) of Leotiomycetes increased from 0.42% to 0.58% to 5.61%. In contrast, in the case of genus *Umbelopsis*, the proportion was reduced in the order of 10.60% to 7.19% to 4.81%. It was also the second largest proportion of genus in each sample after *T. matsutake*, which may be associated with a gradual decrease of 79.01% to 75.22% to 51.98%. As mentioned above, *Umbelopsis* was not a significantly enriched genus in *T. matsutake*-dominant samples, but we suggest that it is positively related to *T. matsutake*. In addition, the genus *Cenococcum* decreased from 1.83% to 1.14% to 0.83% as the seasons changed.

Park and his colleagues conducted in vitro transplantation of *P. densiflora* seedlings inoculated with *T. matsutake* and showed the different results from our study covering several points [54]. Our study did not have a significant change of the transplantation which they conducted, but only observed seasonal changes and regional differences in the wild environment in which *T. matsutake* grows, so there was a clear difference from the in vitro experiment. *Fusarium* was also found very minimal in our sample and *F. oxysporum* was never found. This difference may result from the large environmental difference between the artificially established greenhouse and soils in which *T. matsutake* and its associated fungi have already naturally established their community. They showed as the number of *T. matsutake* decreases, *Suillus* increases. Very little *Suillus* existed in our Sample-1 and we share the same opinion that *Suillus* and *T. matsutake* probably have a negative relationship though the reason is unclear. *Cylindrocarpon pauciseptatum* showed a significantly negative correlation with *T. matsutake* in Park’s work and was also not found in our samples. We assume that it is because of a feature of our sampling area or a wild soil condition of *T. matsutake* and other fungi communities that were formed long ago. The exact reason is not clear, but the difference from the in vitro experiment was evident, and this interesting relationship between these fungi and *T. matsutake* requires further research.

*T. matsutake* is a mushroom that can be found only in autumn when the temperature has cooled. Annual production of *T. matsutake* is determined by the rainfall and temperature in fall [55]. Compared to Gyeongju, the sampling sites at Yeongdeok are affected by the area’s proximity to the ocean. The average daily temperature on the day the sample was collected in Gyeongju was 19.9°C and in Yeongdeok it was 11.5°C [56]. At the time of sampling, the low read number of Yeongdeok samples seems to have been affected by the temperature. The average temperature of October 2018 was 13.7°C, lower than in October 2017 (15.6°C). The precipitation in October 2018 was 223.2 mm per month, a significant increase from October 2017 (49.7 mm per month). The production of *T. matsutake* fruiting body increased rapidly in 2018 due to high rainfall in South Korea [7, 56]. This can be the reason why the ratio of *T. matsutake* in G-F2-2 is higher than in G-F1-2. According to studies by Chung and colleagues, the rate of mycelium occurrence was high in areas with high soil moisture content and this supports the above results [57]. Chung also found that the iron ion content in *T. matsutake* soil was higher than in soil where *T. matsutake* was not dominant. Vaario and his colleagues, however, said that *T. matsutake* did not appear to be related to a specific soil chemistry [14]. On the other hand, pH around the area where *T. matsutake* grows was relatively acidic [14, 57, 58]. However, soil of more than pH 5.0 was also found [59]. Thus, the correlation between *T. matsutake*, the fungal community, and soil chemistry needs to be further studied. Also, soil chemistry and its seasonal changes related to *T. matsutake* need to be measured in further studies in Gyeongju and Yeongdeok soil.

In this study we analyzed the fungal community in the soil under fruiting bodies of *T. matsutake*, and also where the fairy ring of *T. matsutake* was no longer present. We compared it with the fungal community in soil from other regions. In addition, the same spot was investigated three times at intervals of four months at the same spot from fall to the following spring, to observe changes in the soil fungal community related to *T. matsutake*.

We got a high enough number of valid reads in each sample to analyze the fungal community. The valid reads of G-F2-2 were the highest, at 106,048, and those of G-W-1 were the fewest, at 56,395. The OTUs of most samples under fruiting bodies of *T. matsutake* were lower than OTUs from samples where *T. matsutake* was no longer present. This indicates that the fungal diversity is low in soil where *T. matsutake* is dominant. Therefore, the mycelia of *T. matsutake* appear to repress the growth of other fungi and are dominant in the soil fungal community. The soil under *T. matsutake* fruiting bodies was clearly dominated by *T. matsutake* at more than 51%. The difference between where *T. matsutake* was present or not was more than 50%, except in G-S-1 and G-S-2. This implies that when the fruiting body of *T. matsutake* is formed, *T. matsutake* is a dominant species in that spot. In the samples from Yeongdeok, the number of valid reads was slightly lower than that from Gyeongju.

Basidiomycota was the dominant phylum in most samples. The classes in phylum Ascomycota had a tendency to show a smaller percentage in Sample 2 than in Sample 1. *T. matsutake*-dominant G-F1-2 and G-F2-2 samples had only the following genera in common: *Tricholoma*, *Umbelopsis*, *Cenococcum*, *Oidiodendron*, *Sagenomella*, *Cladophialophora*, *Penicillium*, *Rhizopogon*, and *Phialocephala*. In both *T. matsutake*-minor G-F1-1 and G-F2-1, the genera *Sagenomella*, *Umbelopsis*, *Cenococcum*, *Hydnum*, *Russula*, *Oidiodendron*, *Tricholoma*, *Penicillium*, *Sistotrema*, *Phialocephala*, *Talaromyces*, Fungi_uc, Herpotrichiellaceae_uc, and Botryosphaeriaceae_uc were present. In *T. matsutake*-dominant samples, G-F1-2, G-F2-2, and Y-F1-2 had only the genera *Tricholoma*, *Umbelopsis*, *Oidiodendron*, *Sagenomella*, *Cladophialophora*, and *Phialocephala* in common. In *T. matsutake*-minor G-F1-1, G-F2-1, and Y-F1-1, the genera *Umbelopsis*, *Sagenomella*, *Russula*, *Cenococcum*, *Oidiodendron*, *Penicillium*, *Talaromyces*, *Phialocephala*, Herpotrichiellaceae_uc and Fungi_uc were present.

With fewer reads from winter samples, the overall number was reduced, but in the spring, the number of samples and reads increased again. From fall to the following spring, the ratio of phyla Basidiomycota and Mucoromycota gradually dropped. In contrast, the phylum Ascomycota increased. A total of 11 fungal classes were found to represent more than 1% of the total classes in each sample. The classes Leotiomycetes, Eurotiomycetes, Sordariomycetes, Lecanoromycetes, and Mortierellomycetes increased, while Dothideomycetes and Umbelopsidomycetes decreased. The genus *Umbelopsis* accounted for the second-largest proportion of each sample after *T. matsutake*. We suggest that it is positively related to *T. matsutake*.

This study provides a foundation for understanding the ecological relationships between *T. matsutake* and other fungi in soil. However, further studies are necessary to analyze and investigate how *T. matsutake* interacts with other fungi, microorganisms, and roots of *P. densiflora*. Although several authors have published results from similar research on the *T. matsutake*-related microbial community, it was difficult to find commonality because of sampling methods and regional differences. Therefore, it is necessary to find a method that is accurate, uniform, and standardized to analyze the *T. matsutake*-related microbial community.

## Figures and Tables

**Table 1:** Sampling information of ten samples of the soil fungal community related to *T. matsutake*.

	Gyeongju				Yeongdeok
	
	October 2017	October 2018	February 2019	June 2019	November 2017
25~30 cm far from the fairy ring	G-F1-1	G-F2-1	G-W-1	G-S-1	Y-F1-1
Under the fruiting body of *T. matsutake*	G-F1-2	G-F2-2	G-W-2	G-S-2	Y-F1-2

**Table 2:** Pyrosequencing results and statistical analysis of the soil fungal community related to *T. matsutake*.

	G-F1-1	G-F1-2	G-F2-1	G-F2-2	G-W-1	G-W-2	G-S-1	G-S-2	Y-F1-1	Y-F1-2
Number of Valid reads	101347	94695	92141	106048	56395	57412	94346	102565	89952	86224
OTUs^a^	347	203	578	211	554	230	439	469	664	124
Chao1^b^	348.68	204.38	596.83	217.52	561.17	238.12	447.52	475.43	665.60	124.58
Shannon^c^	2.90	2.05	3.69	0.97	3.54	1.25	2.61	2.65	4.01	1.70
Simpson^d^	0.11	0.30	0.06	0.64	0.08	0.58	0.19	0.28	0.04	0.29
Goods lib. Coverage^e^(%)	99.98	99.99	99.92	99.98	99.92	99.94	99.96	99.96	99.98	99.99

^a^OTUs: Operational Taxonomic Units

^b^Chao1: Chao1 estimation for species richness

^c^Shannon: Shannon index for species diversity, > 0, higher, more diverse

^d^Simpson: Simpson index for species diversity, 0 ~ 1, 1 = the simplest

^e^Goods Lib. Coverage: [1 - (number of singleton OTUs / number of total reads)] × 100

**Table 3:** Composition (%) of *T. matsutake* in each sample (using the sum of reads of *Tricholoma matsutake*, *Tricholoma matsutake_1*, and *Tricholoma matsutake_7*).

G-F1-1	G-F1-2	G-F2-1	G-F2-2	G-W-1	G-W-2	G-S-1	G-S-2	Y-F1-1	Y-F1-2
0.00	**57.76**	0.12	**79.13**	0.01	**75.19**	38.92	**52.11**	0.00	**52.25**

**Table 4:** Gradient heatmap of the taxonomic composition (%) at the genus level of each sample for comparison by region (including *T. matsutake*, _uc: unclassified, _g: the genus name was unknown).

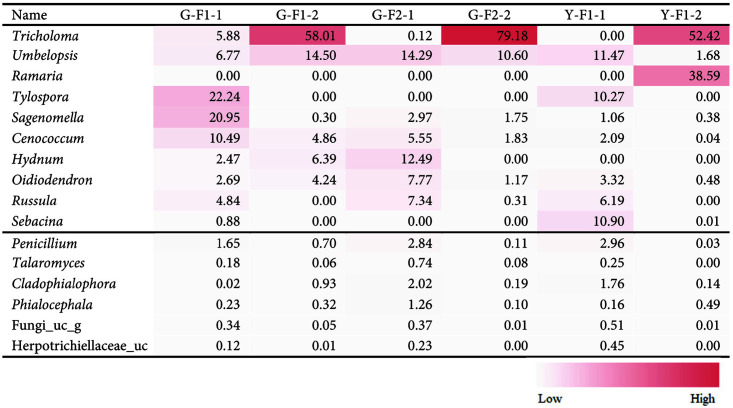

**Table 5:** The number of reads for the common species in G-F1-2, G-F2-2, and Y-F1-2 (_uc: unclassified, _s: the species name was unknown).

Name	G-F1-2	G-F2-2	Y-F1-2
*Umbelopsis dimorpha*	13420	11133	1095
*Oidiodendron chlamydosporicum*	450	347	325
*Oidiodendron*_uc	642	759	26
*Sagenomella diversispora*	34	151	2
*Cenococcum*_uc	4500	52	6
*Sistotrema*_uc	2	15	10
*Cladophialophora*_uc	872	7	115
*Pezicula*_uc	128	7	85
*Oidiodendron pilicola*	657	160	8
Fungi_uc_s	47	8	7
*Umbelopsis*_uc	231	10	379
*Sagenomella*_uc	6	10	24
*Tricholoma*_uc	230	2	125
Basidiomycota_uc_s	26	2	3
